# Identification of ageing-associated gene signatures in heart failure with preserved ejection fraction by integrated bioinformatics analysis and machine learning

**DOI:** 10.1016/j.gendis.2024.101478

**Published:** 2024-12-03

**Authors:** Guoxing Li, Qingju Zhou, Ming Xie, Boying Zhao, Keyu Zhang, Yuan Luo, Lingwen Kong, Diansa Gao, Yongzheng Guo

**Affiliations:** a Department of Cardiology, The First Affiliated Hospital of Chongqing Medical University, Chongqing 400016, China; b Cardiovascular Disease Laboratory of Chongqing Medical University, Chongqing 400016, China; c Department of Health Management Center, Chongqing General Hospital, Chongqing University, Chongqing 400010, China; d Department of Cardiothoracic Surgery, Chongqing Emergency Medical Center, Chongqing University Central Hospital, Chongqing University, Chongqing 400010, China; e Department of Vascular Surgery, The First Affiliated Hospital of Chongqing Medical University, Chongqing 400016, China

**Keywords:** Ageing, Bioinformatics analysis, HFpEF, Immune dysfunction, Machine learning

## Abstract

The incidence of heart failure with preserved ejection fraction (HFpEF) increases with the ageing of populations. This study aimed to explore ageing-associated gene signatures in HFpEF to develop new diagnostic biomarkers and provide new insights into the underlying mechanisms of HFpEF. Mice were subjected to a high-fat diet combined with L-NG-nitroarginine methyl ester (l-NAME) to induce HFpEF, and next-generation sequencing was performed with HFpEF hearts. Additionally, separate datasets were acquired from the Gene Expression Omnibus (GEO) database. The differentially expressed genes (DEGs) were used to identify ageing-related DEGs. Support vector machine, random forest, and least absolute shrinkage and selection operator algorithms were employed to identify potential diagnostic genes from ageing-related DEGs. The diagnostic value was assessed using a nomogram and receiver operating characteristic curve. The gene and related protein expression were verified by reverse transcription PCR and western blotting. The immune cell infiltration in hearts was analysed using the single-sample gene-set enrichment analysis algorithm. The results showed that the merged HFpEF datasets comprised 103 genes, of which 15 ageing-related DEGs were further screened in. The ageing-related DEGs were primarily associated with immune and metabolism regulation. AGTR1a, NR3C1, and PRKAB1 were selected for nomogram construction and machine learning-based diagnostic value, displaying strong diagnostic potential. Additionally, ageing scores were established based on nine key DEGs, revealing noteworthy differences in immune cell infiltration across HFpEF subtypes. In summary, those results highlight the significance of immune dysfunction in HFpEF. Furthermore, ageing-related DEGs might serve as promising prognostic and predictive biomarkers for HFpEF.

## Introduction

Epidemiological investigations indicate that about 50% of the heart failure population has a preserved or normal ejection fraction, namely heart failure with preserved ejection fraction (HFpEF).^1^^,^^2^ Due to the complexities and heterogeneity, the pathophysiological mechanisms underlying HFpEF are still far from elucidation. Consequently, the specific diagnostic biomarkers and evidence-based clinical therapies for HFpEF remain scarce.

Ageing has been identified as a risk factor for HFpEF.^3^ Various studies, including ours, have demonstrated that ageing shares several features, such as insulin resistance, obesity, inflammation, and metabolic dysfunction with HFpEF.^4^, ^5^, ^6^ A previous study demonstrated that a combination of a high-fat diet, desoxycorticosterone pivalate, and ageing recapitulated the typical pathological phenotype of HFpEF,^3^ further supporting that ageing might exhibit some similar pathophysiological mechanisms as HFpEF. Large-scale next-generation sequencing has revealed the genomic landscape of HFpEF and ageing. As similar pathophysiological mechanisms might be associated with genes with similar functions, an integrated analysis of the genome-wide expression profiles of HFpEF and ageing might offer novel insights into the pathogenesis of HFpEF. Moreover, based on multiple pieces of evidence, immune cells play crucial roles both in the physiological process of HFpEF and ageing.^7^, ^8^, ^9^ Therefore, investigating immune infiltration in HFpEF hearts might help understand the pathogenesis of HFpEF.

Machine learning has become a popular approach for uncovering underlying mechanisms, identifying relevant biomarker features, and finding therapeutic targets for various diseases.^10^, ^11^, ^12^ In this study, differentially expressed genes (DEGs) in HFpEF were first identified and an ageing score (A-score) model based on ageing-related DEGs (ARDEGsARDEGs) was established via machine learning. Next, different molecular subtypes of HFpEF were defined and the diagnostic value of the genetic panel for HFpEF was evaluated. Finally, the significant roles of immune cells in the distinct molecular subtypes of HFpEF were explored to offer new insights into the immune molecular mechanisms of HFpEF.

## Methods

### Animal experiments

In accordance with the National Institutes of Health Guidelines for the Use of Laboratory Animals, animal experiments were conducted after obtaining approval from The Chongqing Medical University Committee on Animal Care. Eight-week-old male C57BL/6J mice were administered a 60% high-fat diet and 0.5 g/L L-NG-nitroarginine methyl ester (l-NAME) through drinking water to induce HFpEF as previously described.^4^ The mice were maintained in a standard environment with free access to food and water under a 12-h/12-h light/dark cycle. At the end of the experiments, echocardiography was performed to assess cardiac function, and heart samples and plasma were collected and stored in liquid nitrogen until further analysis.

### Data collection and pre-processing

RNA sequencing analysis was conducted by Applied Protein Technology Co., Ltd., Shanghai, China and designated as the heart failure database. The distribution patterns between HFpEF and control mice were visualised using principal component analysis. Additionally, two more expression profile datasets, namely GSE194151^13^ and GSE184120,^14^ were obtained from the Gene Expression Omnibus database (http://www.ncbi.nlm.nih.gov/geo/). Further details of the collected datasets are presented in Table S1. Then “limma” R package was used to correct the batch effect and the “Deseq2″ R package was used to analyse DEGs (Fig. S1).

Moreover, the GeneCards database was used to search for ageing-related genes using the keyword “ageing”.^15^ The obtained list of the ageing-related genes was further analysed after converting them with homologene packages in R. Additional information about the ageing-related genes is presented in Table S2.

### Identiﬁcation of ARDEGs

Common DEGs were obtained by intersecting the DEGs from the heart failure, GSE194151, and GSE184120 datasets. Next, the ARDEGs were identified by intersecting the common DEGs with ageing-related genes. Subsequently, Gene Ontology (GO) and Kyoto Encyclopaedia of Genes and Genomes (KEGG) pathway enrichment analyses of ARDEGs were conducted using the “clusterProfiler” package, considering adjusted *p*-value < 0.05 and false discovery rate-adjusted *p*-value (q value) < 0.05 as significant. Gene set enrichment analysis (GSEA) and gene set variation analysis (GSVA) were also performed to investigate the differences between HFpEF and control samples using the Molecular Signatures Database-derived gene sets “c2.cp.kegg.v7.4.symbols.gmt” and “c2.cp.all.v2022.1.Hs.symbols.gmt”.^16^ Enriched pathways with adjusted *p*-value <0.05 and false discovery rate-adjusted *p*-value (q value) < 0.05 for GSEA and *p*-value < 0.05 and |log Fold Change| > 0.50 for GSVA were considered statistically significant.

### Western blotting

Heart tissues were homogenized in lysis buffer with a protease inhibitor cocktail (Beyotime, Jiangsu, China). After centrifugation at 12,000 *g* and 4 °C, the supernatant was collected. The protein concertation was measured with a BCA assay. Then samples were mixed with loading buffer and boiled for 10 min. After separating with SDS-PAGE gels, the protein was transferred to the PVDF membrane and then blocked with non-fat milk. Then the membranes were probed with primary antibodies at 4 °C overnight and then corresponding secondary antibodies at room temperature for 90 min. Blots were visualized via horseradish peroxidase assay and images were quantified using Image J.

### Semi-quantitative real-time PCR

Total RNA was extracted from heart tissues with TRIzol reagent (TaKaRa, Shiga, Japan). 1000 ng RNA were reverse-transcribed into cDNA with PrimeScript RT Master Mxia and then real-time PCR were performed with particular primers and 18 S rRNA was used as the housekeeping gene. The results were presented as a fold change to the control.

### Diagnostic model construction with ARDEGs via machine learning

The support vector machine (SVM), random forest (RF), and least absolute shrinkage and selection operator (LASSO) algorithms were employed independently to identify the diagnostic genes from the ARDEGs. RF analysis was performed using the R package “randomForest”^17^ with the parameters “set.seed (234)” and “ntree = 1000″ and $I\left(Xxi\right)=-log_{2}p\left(x_{i}\right)$. LASSO was performed using the R package “glmnet” with parameters “set.seed (500)” and “family = binomial”, based on the ARDEGs in the logistic regression model.^18^ The R package “rm” was used to construct the nomogram^19^ and visualize the interactive relationship of ARDEGs in the diagnostic model with the $\text{riskScore}=\sum_{i}\text{Coefficient}\;\left({\text{hub}\;\text{gene}}_{i}\right)\times \text{messenegr}\;\text{RNA}\;\text{expression}\;\left(\text{hub}\;{\text{gene}}_{i}\right)$. The “ggDCA” package^20^ was used to assess the accuracy and the discriminative ability of the diagnostic model based on ARDEGs. Subsequently, the ARDEGs identified SVM, RF, and LASSO models were intersected to obtain the common ARDEGs. The efficacy of common ARDEGs in diagnosing HFpEF was evaluated by the receiver operating characteristic curve.

### HFpEF subtype identification based on the diagnostic model of ARDEGs

Different HFpEF subtypes (cluster 1/cluster 2) in the GSE194151 dataset were identified based on the expression of ARDEGs using the “ConsensusClusterPlus” package^21^ in R. The parameters used for this analysis were maxK = 8, reps = 50, pItem = 0.8, pFeature = 1, clusterAlg = pam, and distance = spearman. The “Deseq2” package was used to determine the DEGs between cluster 1 and cluster 2. The DEGs were selected based on the criteria of |log Fold Change| >0.5 and adjusted *p*-value <0.05 for further analysis. The results were visualized by plotting volcanoes using the “ggplot2” package in R.

### Construction of A-scores

The DEGs and ARDEGs in the GSE194151 dataset were intersected to obtain the screened-in DEGs. Phenotypic scoring calculations were performed to identify the potential mechanism of action and related biological characteristics and pathways of the screened-in DEGs in HFpEF. For this purpose, the single-sample GSEA (ssGSEA) algorithm was used to quantify the relative abundance of each gene in the database. The “GSVA” package^22^ in R was used to calculate the ageing phenotypic scores (A-scores) of each sample in the GSE194151 dataset based on the DEG expression to identify the potential mechanism of action of the common ARDEGs in HFpEF, as well as related biological features and pathways. The A-scores were grouped by median to determine the diagnostic effect of A-scores on the HFpEF model via the receiver operating characteristic curve.

### Immune cell infiltration and correlation analysis

The ssGSEA algorithm was used to quantify the relative abundance of the infiltration of each immune cell.^23^ The enrichment score of the samples in the GSE194151 dataset was calculated using the ssGSEA algorithm in the “GSVA” package. Boxplots were used to display the differences in infiltration abundance of 28 immune cells between the high and low risk score groups determined by LASSO regression, different HFpEF subtypes (cluster 1/cluster 2), and high/low A-scores. Additionally, the correlations between different immune cells within the high/low risk score groups, cluster 1/cluster 2, and high/low A-scores were calculated using Spearman's correlation and visualized using the “ggplot2” R package.

### The regulatory network of immune cells and ARDEGs

The gene expression matrix of GSE194151 dataset was combined to calculate the correlation between immune cells and ARDEGs in different groups to analyze the regulatory network of ARDEG expression and immune cells. The results were visualized using the “ggplot2” R package. In addition, the peripheral blood mononuclear cell data from HFpEF patients (GSE223527) were downloaded to investigate the ARDEG expression in different immune cells.

### Statistical analysis

R (Version 4.2.2) was used for statistical analyses. The Wilcoxon test was used to evaluate the expression differences between the two groups. The Kruskal–Wallis test was used for comparisons involving three or more groups. The logistic regression algorithm was used to develop the predictive model, and the diagnostic accuracy of the model was measured using a receiver operating characteristic curve. Unless specified, Spearman correlation analysis was used to calculate the correlation coefficient between different variables, and all statistical *p*-values were two-tailed. Statistical significance was set at *p* < 0.05.

## Results

### High-fat diet and l-NAME induced HFpEF phenotype in mice

As shown in Figure S2A–C, the combination of a high-fat diet and l-NAME showed no significant alteration in left ventricular systolic function (Fig. S2B) but significantly reduced the diastolic dysfunction (Fig. S2C) and exercise tolerance (Fig. S2D) in mice. Moreover, the lung edema (Fig. S2E) and brain natriuretic peptide (Fig. S2F) levels significantly increased in mice. Then, hearts were excised for bulk RNA sequencing. The principal component analysis showed pronounced discrimination between hearts from two groups (Fig. S3A) and the DEGs were displayed in Figure S3B. Moreover, the KEGG analysis revealed that the DEGs were primarily enriched in the metabolism pathway, consistent with the clinical features of HFpEF (Fig. S3C). Those results showed that the combination of a high-fat diet and l-NAME successfully induced a periclinal HFpEF mouse model.

### Identification of DEGs and ARDEGs

**Figure 1:**
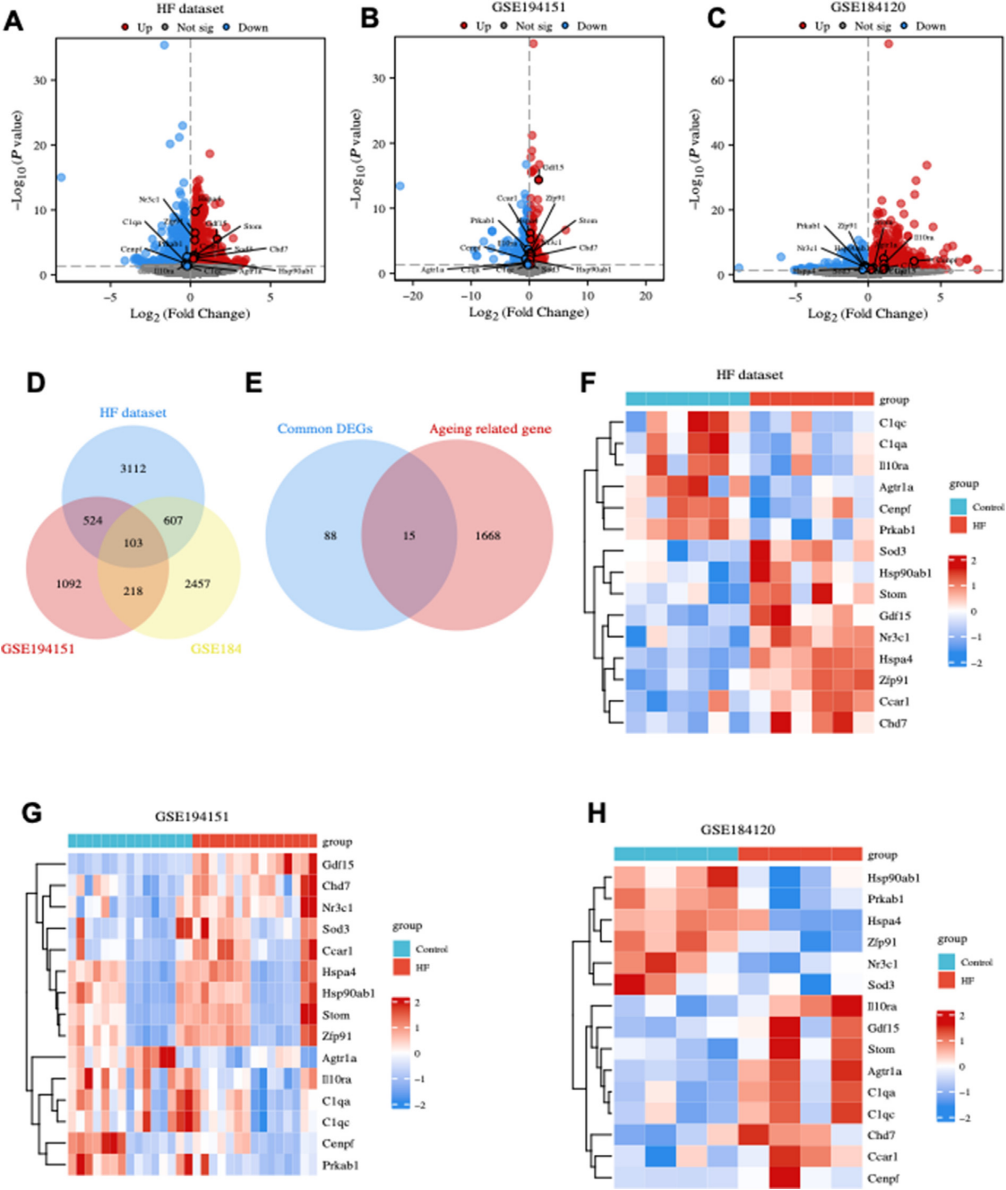
Identification of differentially expressed genes (DEGs) and ageing-related DEGs (ARDEGs). **(A**–**C)** The volcano plot of the DEGs in our dataset (heart failure dataset) (A), GSE194151 dataset (B), and GSE184120 dataset (C). **(D)** Identification of the common DEGs. **(E)** Identification of the ARDEGs. **(F–H)** Heatmap of ARDEGs in the heart failure (F), GSE194151 (G), and GSE184120 (H) datasets.

Based on our dataset, 2345 genes were significantly up-regulated and 1992 genes were significantly down-regulated in HFpEF hearts compared with control hearts (Fig. 1A). Similarly, the GSE194151 dataset revealed 975 significantly up-regulated genes and 962 significantly down-regulated genes in the HFpEF group compared with the control group (Fig. 1B). Furthermore, the GSE184120 dataset depicted 1997 up-regulated genes and 1388 down-regulated genes in the HFpEF group compared with the control group (Fig. 1C). These DEGs were intersected to identify the common DEGs, which resulted in 103 genes, as illustrated in Figure 1D.

The common DEGs and ageing-related genes were intersected to identify ARDEGs, which resulted in the identification of 15 ARDEGs (Fig. 1E). The expression of ARDEGs in the heart failure (Fig. 1F), GSE194151 (Fig. 1G), and GSE184120 (Fig. 1H) datasets were visualized using heatmap. GO enrichment analysis revealed that ARDEGs were primarily enriched in various processes such as “striated muscle tissue development”, “superoxide metabolic process”, “collagen trimer”, and “ATP hydrolysis activity” (Fig. S4A–D). Additionally, the KEGG analysis revealed that ARDEGs were primarily enriched in “coronavirus disease 2019”, “antigen processing and presentation”, “complement and coagulation cascades”, “apelin signaling pathway”, and “lipid and atherosclerosis pathway” (Fig. S4E, F).

GSEA (Fig. S5) and GSVA (Fig. S6) were conducted on the GSE194151 dataset, and the results indicated that the ARDEGs were primarily associated with immune response and metabolic pathways, both of which play crucial roles in the pathogenesis of HFpEF and ageing.

Moreover, with a single-cell dataset from HFpEF patients (GSE223527), we identified six different types of immune cells in peripheral blood mononuclear cells as shown in Figure S7A and B. Further analysis revealed that the proportion of neutrophils and CD8 T cells in HFpEF patients was significantly increased, however, the proportion of CD4 T cells decreased (Fig. S7C). Together with that, the ARDEGs were expressed in all identified immune cells, especially in neutrophils (Fig. S7D). Those data all suggested that the ARDEGs might play an important role in immune response in HFpEF.

### Diagnostic biomarker identification and verification via machine learning

**Figure 2:**
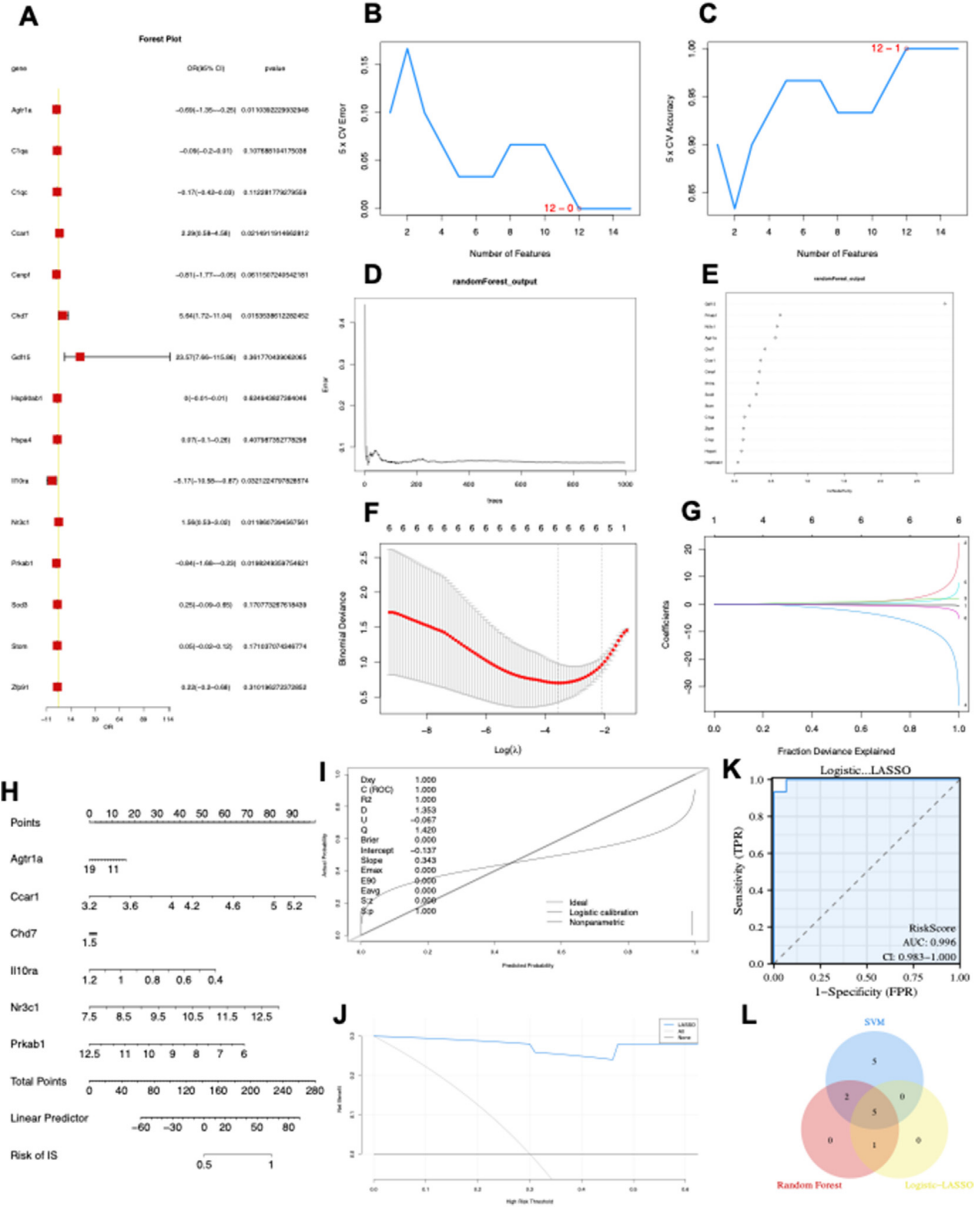
Diagnostic biomarker identification and verification based on ARDEGs via machine learning. **(A)** The forest plot illustrating ARDEG expression via the logistic regression analysis. **(B, C)** The number of genes with the lowest error rate (B) and the highest accuracy rate (C) in the SVM model. **(D, E)** Random forest analysis was conducted to analyse the ARDEGs and extract potential diagnostic biomarkers. **(F, G)** Biomarker screening via LASSO regression analysis. **(H–K)** The visible nomogram for diagnosis (H), and the diagnostic value evaluation (I–K). **(L)** The Venn diagram showing five candidate diagnostic genes identified via SVM, logic-LASSO, and random forest algorithms. SVM, support vector machine; ARDEGs, ageing-related differentially expressed genes; LASSO, least absolute shrinkage and selection operator; ROC, receiver operating characteristic; AUC, the area under the ROC curve; DCA, decision curve analysis.

The forest plot depicting the 15 ARDEGs is presented in Figure 2A. Using the SVM algorithm, it was found that the SVM model had the highest accuracy when 12 genes were used (Fig. 2B, C). Subsequently, the RF algorithm was employed to extract potential diagnostic biomarkers (Fig. 2D, E). Using the LASSO regression algorithm, six potential candidate biomarkers were identified, as presented in Figure 2F and G. The nomogram indicated the importance of each gene in the ARDEG diagnostic model (Fig. 2H). The accuracy of the ARDEG diagnostic model was evaluated using the calibration analysis, which showed high accuracy in diagnosing diseases, as demonstrated in Figure 2I and J. Furthermore, the area under the receiver operating characteristic curve (AUC) value in the GSE194151 dataset was 0.996, indicating the strong diagnostic performance of the ARDEG diagnostic model for HFpEF (Fig. 2K). Finally, the intersection of genes from the SVM, RF, and LASSO regression was visualized using a Venn diagram (Fig. 2L). Five common ARDEGs, namely angiotensin II receptor type 1 a (AGTR1a), cell division cycle and apoptosis regulator 1 (CCAR1), interleukin receptor 10 subunit alpha (Il10RA), nuclear receptor subfamily 3 group C member 1 (NR3C1), and 5′-adenosine monophosphate-activated protein kinase subunit beta-1 (PRKAB1), were identified for the final validation.

**Figure 3:**
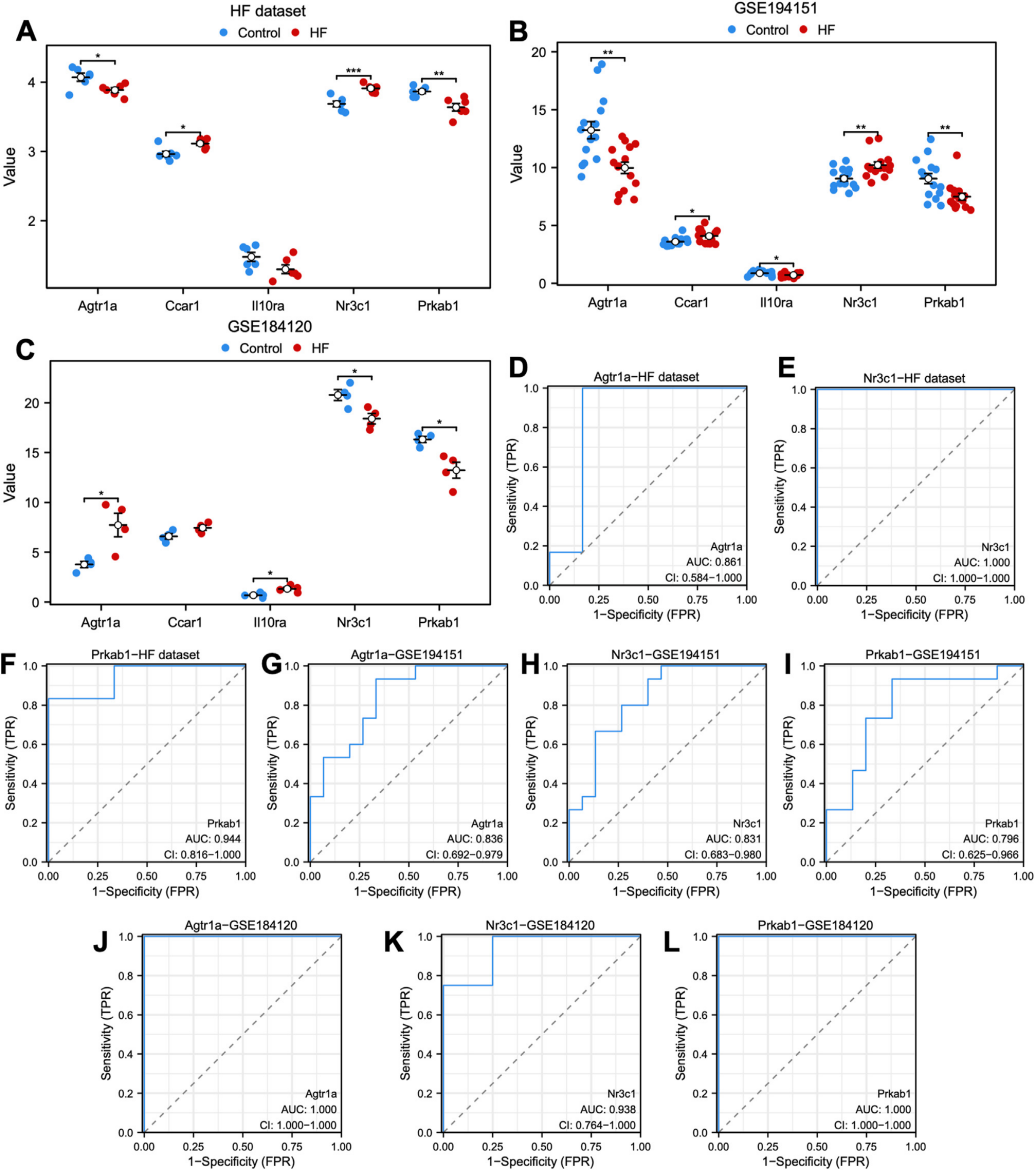
The expression of five candidate diagnostic genes and the verification of diagnostic specificity and sensitivity. **(A**–**C)** The expression of candidate diagnostic genes in heart failure (A), GSE194151 (B), and GSE184120 datasets (C). **(D**–**L)** The ROC curve of each candidate gene (Agtr1a, NR3C1, and PRKAB1) in the heart failure (D–F), GSE194151 (G–I), and GSE184120 datasets (J–L). Not significant, *p* ≥ 0.05; ∗*p* < 0.05, ∗∗*p* < 0.01, ∗∗∗*p* < 0.001. HFpEF, heart failure with preserved ejection fraction; ROC, receiver operating characteristic; AUC, the area under the curve.

The specific expression levels of the five common ARDEGs were compared between HFpEF and control groups using the Wilcoxon rank sum test in heart failure (Fig. 3A), GSE194151 (Fig. 3B), and GSE184120 (Fig. 3C) datasets. AGTR1a, NR3C1, and PRKAB1 exhibited significant statistical differences in all the three datasets. The receiver operating characteristic curves were then constructed to assess the diagnostic specificity and sensitivity of each gene in the three datasets. In the heart failure dataset (Fig. 3D–F), AGTR1a (AUC: 0.861), NR3C1 (AUC: 1.000), and PRKAB1 (AUC: 0.944) demonstrated significant diagnostic value. In the GSE184120 dataset (Fig. 3G–I), AGTR1a (AUC: 0.836), NR3C1 (AUC: 0.831), and PRKAB1 (AUC: 0.796) showed diagnostic value. In the GSE184120 dataset (Fig. 3J–L), AGTR1a (AUC: 1.000), NR3C1 (AUC: 0.938), and PRKAB1 (AUC: 1.000) exhibited high diagnostic value for HFpEF.

**Figure 4:**
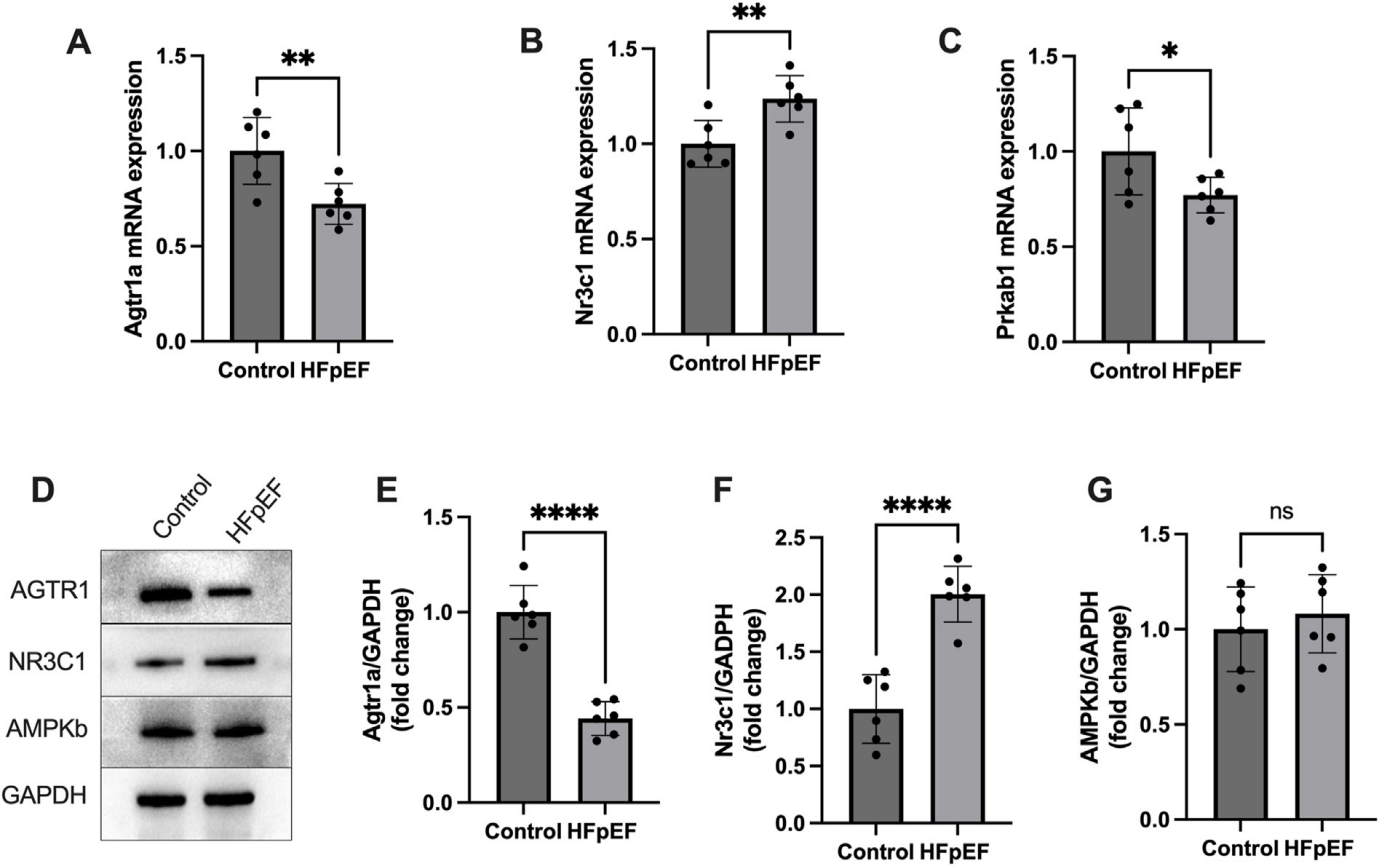
The mRNA and protein levels of Agtr1, Nr3c1, and Prkab1 in hearts from HFpEF mice were verified. **(A**–**C)** The mRNA expression of Agtr1, Nr3c1, and Prkab1 in HFpEF hearts. **(D**–**G)** The levels of protein encoded by Agtr1, Nr3c1, and Prkab1 in HFpEF hearts. Not significant, *p* ≥ 0.05; ∗*p* < 0.05, ∗∗*p* < 0.01. HFpEF, heart failure with preserved ejection fraction.

Moreover, we verified the mRNA expression of Agtr1a, Nr3c1, and Prkab1 in HFpEF hearts. Results showed a significantly increased mRNA expression of Nr3c1, but a reduced mRNA expression of Agtr1a and Prkab1 in HFpEF hearts (Fig. 4A–C). In addition, we measured the protein level encoded by those three genes in HFpEF hearts. Results showed that AGTR1 protein level reduced, NR3C1 protein level increased, and the protein levels of AMPKb had no significant change in HFpEF hearts (Fig. 4D–G).

Collectively, these findings suggested that all three candidate genes could serve as potential diagnostic markers for HFpEF, and AGTR1 and NR3C1 might be involved in the progression of HFpEF.

### Immune cell infiltration analysis based on the ARDEG diagnostic model

**Figure 5:**
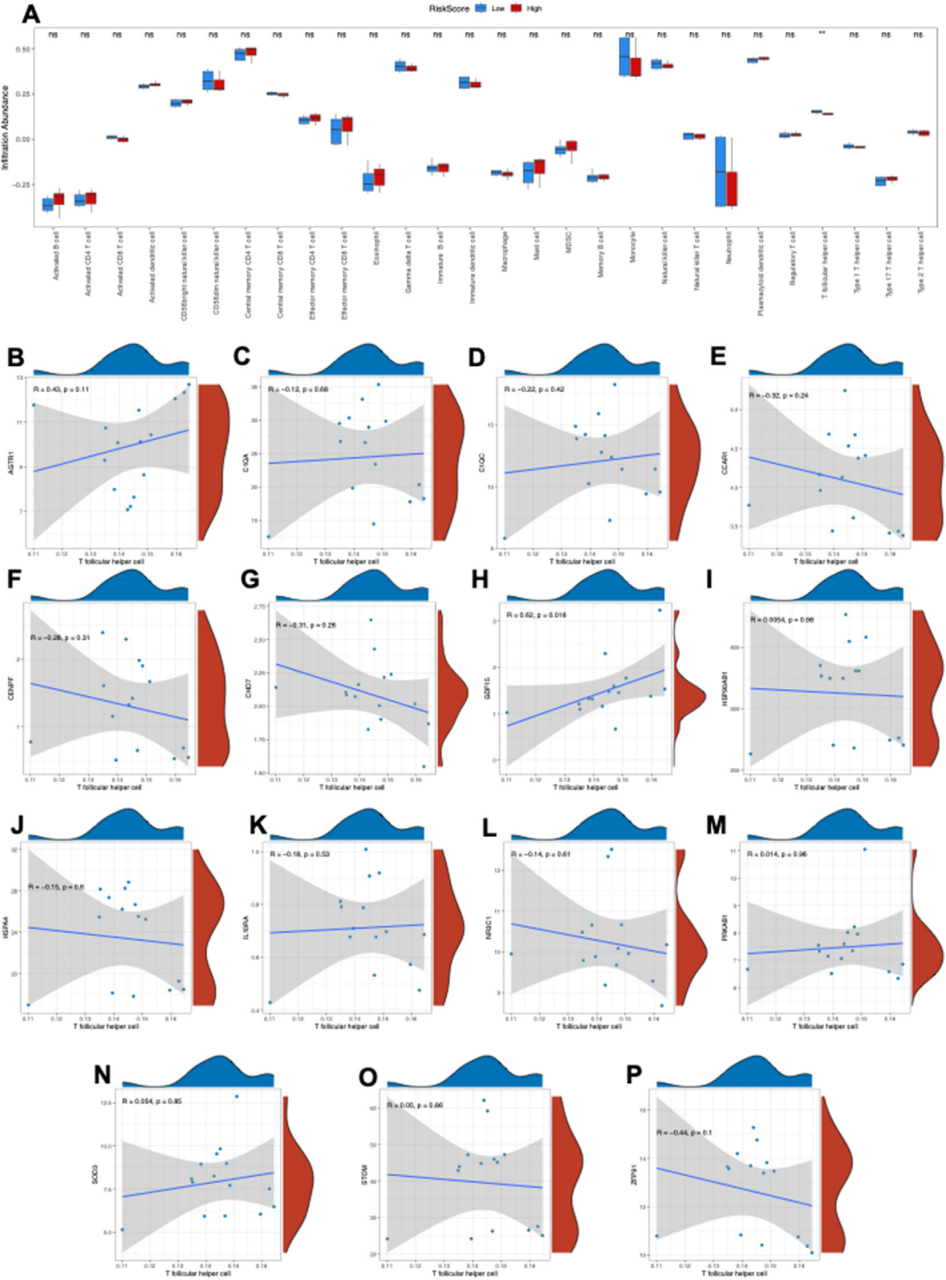
Immune cell infiltration analysis between the low risk score and high risk score groups based on the ARDEG diagnostic model. **(A)** The proportion of 28 immune cell types in the low risk score group and high risk score group visualized by the bar plot. **(B–P)** The correlation of T follicular helper cell infiltration and ARDEGs was analyzed via the Spearman algorithm. Not significant, *p* ≥ 0.05; ∗*p* < 0.05, ∗∗*p* < 0.01, ∗∗∗*p* < 0.001. HFpEF, heart failure with preserved ejection fraction; ssGSEA, single-sample gene set enrichment analysis; ARDEG, ageing-related differentially expressed gene.

The ssGSEA method was used to explore the immune cell infiltration in HFpEF based on the ARDEG diagnostic model. First, HFpEF samples in the GSE194151 dataset were categorized into high and low risk score groups based on the risk score value in the ARDEG diagnostic model. Then a bar plot was used to visualize the 28 immune cell types in the high and low risk score groups. The results revealed that only the T follicular helper cell level was significantly higher in the low risk score group than high risk score group (Fig. 5A). Additionally, the correlation between T follicular helper cell infiltration and ARDEGs was analyzed using the Spearman algorithm (Fig. 5B–P). The findings revealed that growth/differentiation factor 15 (GDF15) was positively correlated with the level of T follicular helper cell infiltration (Fig. 5H).

### HFpEF subtype identification based on the ARDEG diagnostic model

**Figure 6:**
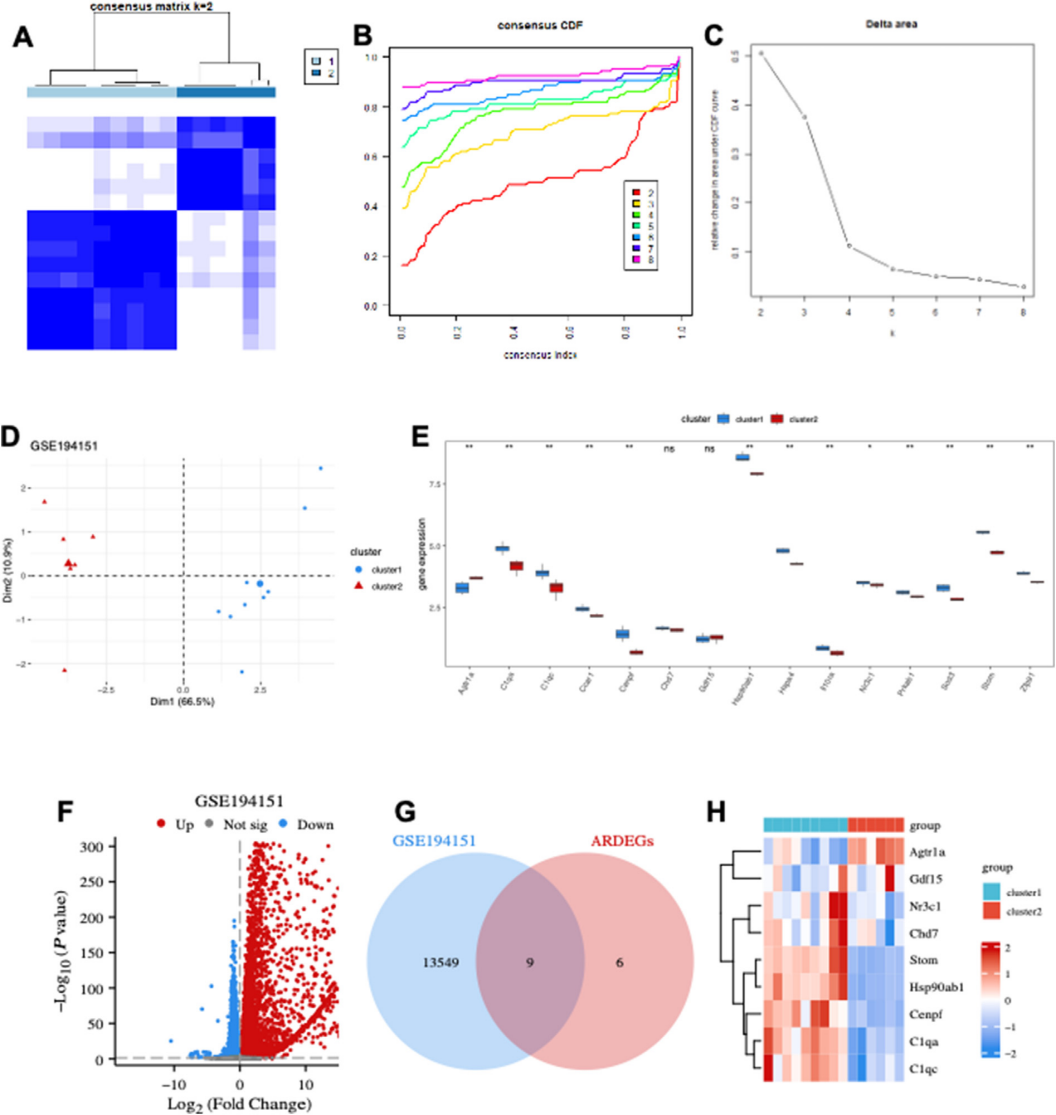
HFpEF subtype identification based on the ARDEG diagnostic model. **(A)** The heatmap exhibiting the two HFpEF clusters with *k* = 2 based on the ARDEGs. **(B)** Cumulative distribution function (CDF) for *k* = 2–9. **(C)** Delta diagram illustrating the variations of the area under the CDF curve for *k* = 2–9. **(D)** PCA based on the results of the consensus clustering analysis. **(E)** ARDEG expressions in two different HFpEF clusters. **(F)** Volcano plot of the DEGs in two different HFpEF clusters. **(G)** Venn diagram of nine key DEGs identified by intersecting the DEGs and ARDEGs. **(H)** The heatmap exhibiting expression of the key DEGs in two different HFpEF clusters. HFpEF, heart failure with preserved ejection fraction; CDF, cumulative distribution function; PCA, principal component analysis; DEGs, differentially expressed genes; ARDEGs, ageing-related DEGs.

The potential of ARDEGs as diagnostic markers for HFpEF was explored and consensus clustering was performed using the “ConsensusClusterPlus” algorithm on the GSE194151 dataset based on the ARDEG diagnostic model. The analysis revealed two distinct HFpEF subtypes (cluster 1 and cluster 2) (Fig. 6A), which were identified based on consensus cumulative distribution function (Fig. 6B) and delta area plots (Fig. 6C). A pronounced discrimination was observed between cluster 1 and cluster 2 (Fig. 6D). The expression of ARDEGs was significantly different between the two clusters, except for chromodomain helicase deoxyribonucleic acid binding protein 7 (CHD7) and GDF15 (Fig. 6E). A total of 13,558 DEGs, including 7190 up-regulated and 6368 down-regulated genes, were identified (Fig. 6F). Venn diagram analysis revealed that nine key DEGs, namely AGTR1a, centromere protein F (CENPF), stomatin (STOM), NR3C1, GDF15, CHD7, complement C1qA chain (C1QA), heat-shock protein, 90-KD, alpha, class B, member 1 (HSP90AB1), and complement C1qC chain (C1QC), overlapped with ARDEGs (Fig. 6G). The heatmap indicated that the expression of these nine key DEGs was significantly different between cluster 1 and cluster 2 (Fig. 6H), supporting the effectiveness of the subtype identification on HFpEF based on the ARDEG diagnostic model.

### Immune cell infiltration analysis based on HFpEF subtypes

**Figure 7:**
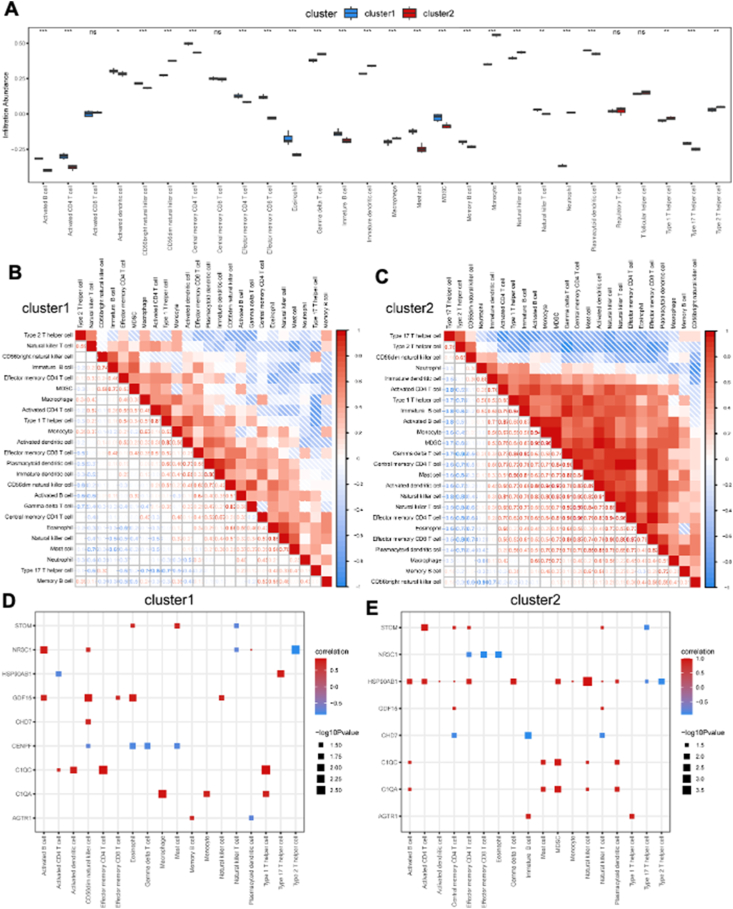
Immune cell infiltration analysis between two different HFpEF clusters. **(A)** The proportion of 28 immune cell types in two different HFpEF clusters (cluster 1 and cluster 2). **(B, C)** Correlation of 24 immune cell types with a significantly different infiltration abundance in cluster 1 (B) and cluster 2 (C). **(D, E)** The correlation of 24 immune cell types with a significantly different infiltration abundance and key DEGs in cluster 1 (D) and cluster 2 (E). HFpEF, heart failure with preserved ejection fraction; ssGSEA, single-sample gene set enrichment analysis; DEGs, differentially expressed genes.

The ssGSEA algorithm was used to investigate the differences in immune cell infiltration between cluster 1 and cluster 2. The proportions of 24 types of immune cells were compared between the two clusters using bar plots, and the results revealed significant differences (Fig. 7A). Specifically, cluster 1 had higher levels of activated B cells, activated CD4 T cells, activated dendritic cells, CD56 bright natural killer cells, central memory CD4 T cells, effector memory CD4 T cells, effector memory CD8 T cells, eosinophils, immature B cells, macrophages, mast cells, myeloid-derived suppressor cells, memory B cells, natural killer T cells, plasmacytoid dendritic cells, and type 17 T helper cells but lower levels of CD56 dim natural killer cells, gamma delta T cells, immature dendritic cells, monocytes, natural killer cells, neutrophils, type 1 T helper cells, and type 2 T helper cells (Fig. 7A). In cluster 1, there was a high positive correlation between immature dendritic cells and plasmacytoid dendritic cells (Fig. 7B), while in cluster 2, the highest positive correlation was observed between effector memory CD4 T cells and effector memory CD8 T cells (Fig. 7C). Additionally, correlation analysis revealed significant positive correlations between the content of immune infiltration and the key DEGs in cluster 1 (Fig. 7D) and cluster 2 (Fig. 7E).

### Construction of A-scores

**Figure 8:**
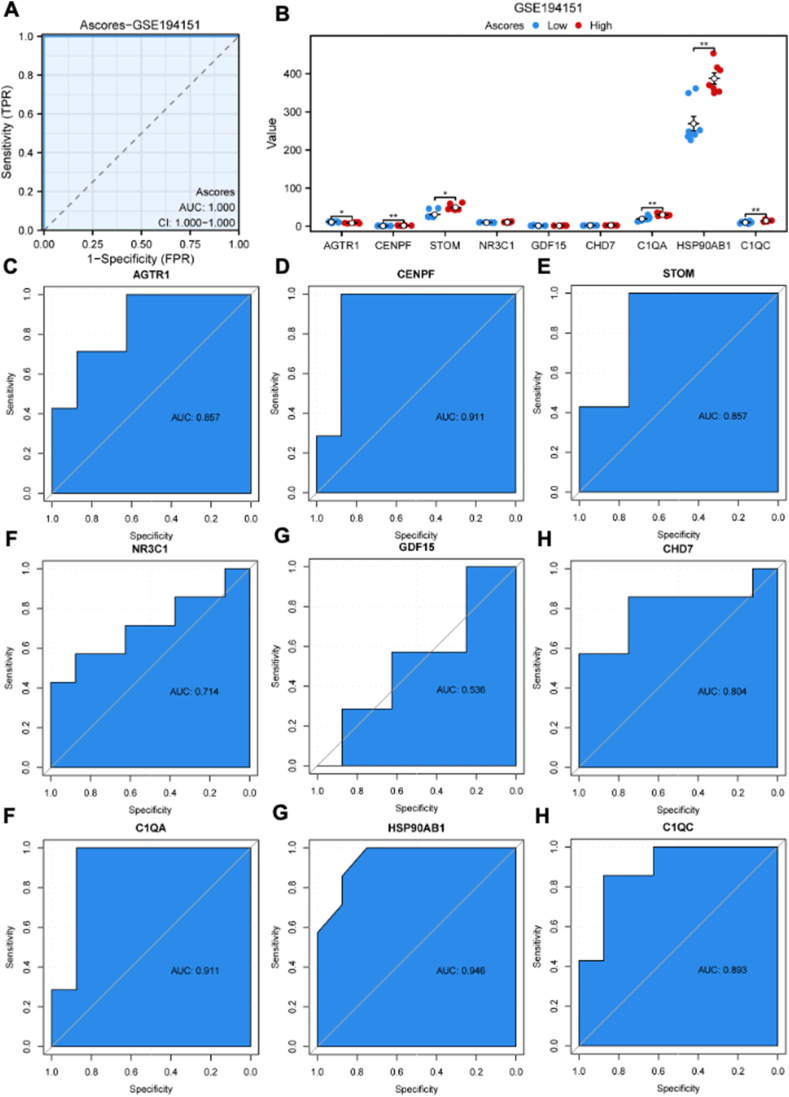
Construction of A-scores and the verification of diagnostic specificity and sensitivity. **(A)** The ROC curve of A-scores for the GSE194151 dataset. **(B)** The expression of key DEGs in the high and low A-score groups. **(C–K)** The ROC curve of each key DEG shows the diagnostic value for the HFpEF subtypes based on the A-scores. Not significant, *p* ≥ 0.05; ∗*p* < 0.05, ∗∗*p* < 0.01, ∗∗∗*p* < 0.001. HFpEF, heart failure with preserved ejection fraction; DEGs, differentially expressed genes; A-scores, ageing scores; KM, Kaplan–Meier; ROC, receiver operating characteristic; AUC, area under the ROC curve.

ssGSEA was used to construct A-scores based on the nine key DEGs. The receiver operating characteristic curve analysis revealed that the AUC value was 1.000, indicating high diagnostic specificity and sensitivity of A-scores for HFpEF in the GSE194151 dataset (Fig. 8A). Based on A-scores, HFpEF samples were categorized into high and low A-score groups. The bar plot indicated that the high A-score group had a higher expression of CENPF, STOM, C1QA, HSP90AB1, and C1QC and a lower expression of AGTR1 and CENPF (Fig. 8B). Further, the diagnostic specificity and sensitivity of each gene to different HFpEF subtypes based on A-scores were evaluated using the receiver operating characteristic curve (Fig. 8C–K). The AUC values for AGTR1, CENPF, STOM, NR3C1, GDF15, CHD7, C1QA, HSP90AB1, and C1QC were 0.857, 0.911, 0.857, 0.714, 0.536, 0.804, 0.911, 0.946, and 0.893, respectively. These results suggested that CENPF, C1QA, and HSP90AB1 have a high diagnostic value for HFpEF subtypes based on A-scores.

### Immune cell infiltration analysis based on A-scores

**Figure 9:**
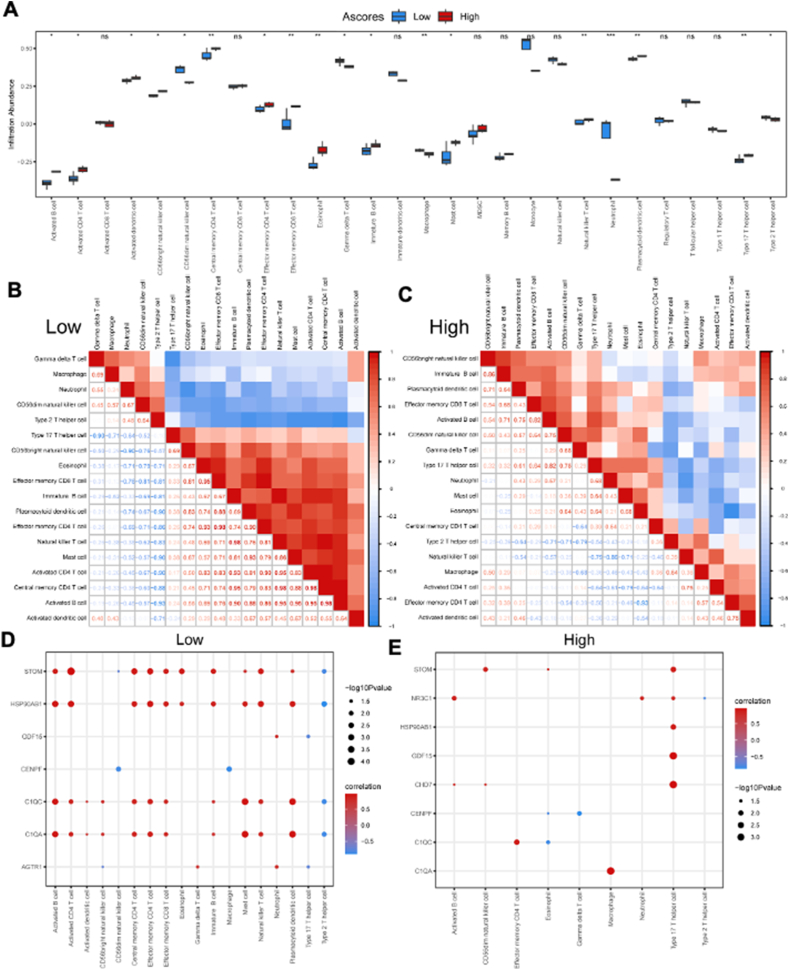
Immune cell infiltration analysis between the high and low A-score groups. **(A)** The proportion of 28 immune cell types in the high and low A-score groups. **(B, C)** Correlation of 18 immune cell types with a significantly different infiltration abundance in the low (B) and high (C) A-score groups. **(D, E)** The correlation of 18 immune cell types with a significantly different infiltration abundance and key DEGs in the high and low A-score groups. Not significant, *p* ≥ 0.05; ∗*p* < 0.05, ∗∗*p* < 0.01, ∗∗∗*p* < 0.001. HFpEF, heart failure with preserved ejection fraction; ssGSEA, single-sample gene set enrichment analysis; A-score, ageing score; DEGs, differentially expressed genes.

The ssGSEA algorithm was used to investigate the difference in immune cell infiltration between the high and low A-score groups. Results revealed that the high and low A-score groups exhibited significant differences in the proportion of 24 types of immune cells (Fig. 9A). The bar plot revealed that the high A-score group had higher levels of activated B cells, activated CD4 T cells, activated dendritic cells, CD56 bright natural killer cells, central memory CD4 T cells, effector memory CD4 T cells, effector memory CD8 T cells, eosinophils, immature B cells, mast cells, natural killer T cells, plasmacytoid dendritic cells, and type 17 T helper cells and lower levels of CD56 dim natural killer cells, gamma delta T cells, macrophages, neutrophils, and type 2 T helper cell (Fig. 9A). The correlation analysis revealed that the highest correlation in the low A-score group was observed between effector memory CD4 T cells and effector memory CD8 T cells, as well as between natural killer T cells and immature B cells (Fig. 9B). However, in the high A-score group, most immune cells exhibited a negative correlation, except for effector memory CD4 T cells and eosinophils (Fig. 9C). Furthermore, the correlation analysis revealed a significant correlation between the content of immune infiltration and the key DEGs. In the low A-score group, a positive correlation was predominant (Fig. 9D). In contrast, in the high A-score group, only nine immune cell types showed a correlation with the key DEGs, and a positive correlation was predominant (Fig. 9E).

## Discussion

HFpEF is a complex clinical syndrome with high morbidity and mortality^24^ and has been suggested to be more common among the elderly population.^25^ However, the molecular mechanisms underlying HFpEF are not yet fully understood due to the lack of pro-clinic models. Recently, researchers have developed an HFpEF model by combining a high-fat diet and an endothelial nitric oxide synthase inhibitor l-NAME, which mimics the typical pathological features of HFpEF in mice,^26^^,^^27^ providing a powerful tool for the pathophysiological research of HFpEF. Additionally, next-generation sequencing has been widely used to explore the mechanisms of HFpEF and has provided valuable information that might aid in identifying diagnostic and therapeutic targets for HFpEF.

HFpEF shares some pathological features with ageing.^28^ Therefore, we identify common DEGs between ageing and HFpEF via integrated bioinformatics analysis and machine learning methods. Five common ARDEGs (AGTR1a, CCAR1, Il10RA, NR3C1, and PRKAB1) were identified. Among them, AGTR1a, CCAR1, Il10RA, and NR3C1 are all involved in immune regulation, indicating the vital role of immune dysfunction in the pathophysiological process of HFpEF. AGTR1 encodes AT1 receptor A and has been reported to be associated with cellular ageing, inflammation, and hypertension according to previous studies.^29^, ^30^, ^31^ Moreover, increased AGTR1a expression is associated with an ageing-like phenotype and higher mortality rates in mice with myocardial infarction,^32^^,^^33^ further confirming the important role of AGTR1a both in ageing and cardiovascular diseases. Additionally, PRKAB1 encodes the non-catalytic subunit of adenosine monophosphate-activated protein kinase, which is a key molecular in regulating fatty acid and blood glucose utilization.^34^^,^^35^ As metabolic dysfunction is a feature of ageing and HFpEF, AGTR1 has an important role in the development of HFpEF. Notably, the expression of ARDEGs in sinoatrial node tissue (GSE184120) differed from that in heart tissue (GSE194151 and our heart failure dataset). That difference suggests that sinoatrial node tissue and heart tissue undergo different pathophysiological mechanisms in HFpEF, although further studies are necessary to confirm this finding.

In our previous study,^4^ it was observed that metabolic dysfunction and oxidative stress were implicated in the progression of HFpEF, both of which are associated with ageing.^36^^,^^37^ In the present study, through bioinformatics analysis, it was identified that the ARDEGs in HFpEF were enriched in “superoxide metabolic process” and “ATP hydrolysis activity”, consistent with our earlier findings. Additionally, inflammation is also a main feature of HFpEF,^38^^,^^39^ and it was observed that ARDEGs were enriched in “antigen processing and presentation”, “complement and coagulation cascades”, and “apelin signaling pathway”, further confirming the crucial roles of immune response in ageing and HFpEF. Consequently, immune cell infiltration analysis showed that the T follicular helper cell level was significantly higher in the low risk score group than in the high risk score group. T follicular helper cells, as a specialized subset of CD4^+^ T cells, play crucial roles in regulating antibody responses and providing protection against foreign pathogens.^40^ These findings suggest that increased T follicular helper cells may alleviate inflammation induced by foreign pathogens and thus protect against HFpEF. However, further research is necessary to confirm this hypothesis.

The receiver operating characteristic curve analysis revealed that AGTR1a, NR3C1, and PRKAB1 exhibited high diagnostic value for HFpEF, and CENPF, C1QA, and HSP90AB1 had a high diagnostic potential for HFpEF subtypes based on A-scores. Additionally, HFpEF was stratified into high and low A-score groups and immune cell infiltration analysis revealed significant differences in the proportion of 24 immune cell types between the two groups. Notably, the key DEGs were positively correlated with the immune infiltration content, suggesting their important role in immune regulation in HFpEF. Although, the precise mechanism of the key DEGs in immune infiltration regulation remains unexplored, targeting them may provide a promising approach for treating HFpEF.

Our study has certain limitations. First, despite pooling three different HFpEF datasets, including our dataset, the total sample size was still relatively small. Therefore, the findings of the present study need to be confirmed with larger studies. Second, although an association was observed between the key DEGs and immune cells, the exact mechanisms by which these key DEGs modulate the immune system still require further investigation. Moreover, whether the A-scores or ARDEGs have prognostic value in HFpEF warrants validation in larger cohorts with comprehensive clinical data.

In conclusion, our study suggests that ARDEGs may serve as promising prognostic and predictive biomarkers for HFpEF and highlight the important role of immune regulation in HFpEF, which might contribute to the identification of therapeutic targets.

## Ethics declaration

All animal experiment designs were approved by the Chongqing Medical University Committee on Animal Care.

## Funding

This work was supported by grants from the National Natural Science Foundation of China (No. 82070238), the Natural Science Foundation of Chongqing, China (No. CSTB2022NSCQ-MSX0913, CSTB2023NSCQ-MSX0219, CSTC2020JCYJ-MSXMX0290), the Chongqing Education Committee of China (No. KJQN202300480), and the Program for Youth Innovation in Future Medicine, Chongqing Medical University (No. W0168).

## CRediT authorship contribution statement

**Guoxing Li:** Formal analysis, Investigation, Writing – original draft. **Qingju Zhou:** Formal analysis, Writing – original draft. **Ming Xie:** Formal analysis, Writing – original draft. **Boying Zhao:** Formal analysis. **Keyu Zhang:** Formal analysis, Visualization. **Yuan Luo:** Formal analysis, Visualization. **Lingwen Kong:** Conceptualization, Supervision. **Diansa Gao:** Formal analysis, Writing – review & editing. **Yongzheng Guo:** Conceptualization, Funding acquisition, Supervision, Writing – review & editing.

## Conflict of interests

The authors declared no competing interests.
