# Supplementary material for: Identification of ageing-associated gene signatures in heart failure with preserved ejection fraction by integrated bioinformatics analysis and machine learning
Source: Genes Dis. 2024 Dec 3;12(4):101478. doi: 10.1016/j.gendis.2024.101478 (PMC12053710; doi:10.1016/j.gendis.2024.101478)
Supplement: Multimedia component 1 [file mmc1.docx]

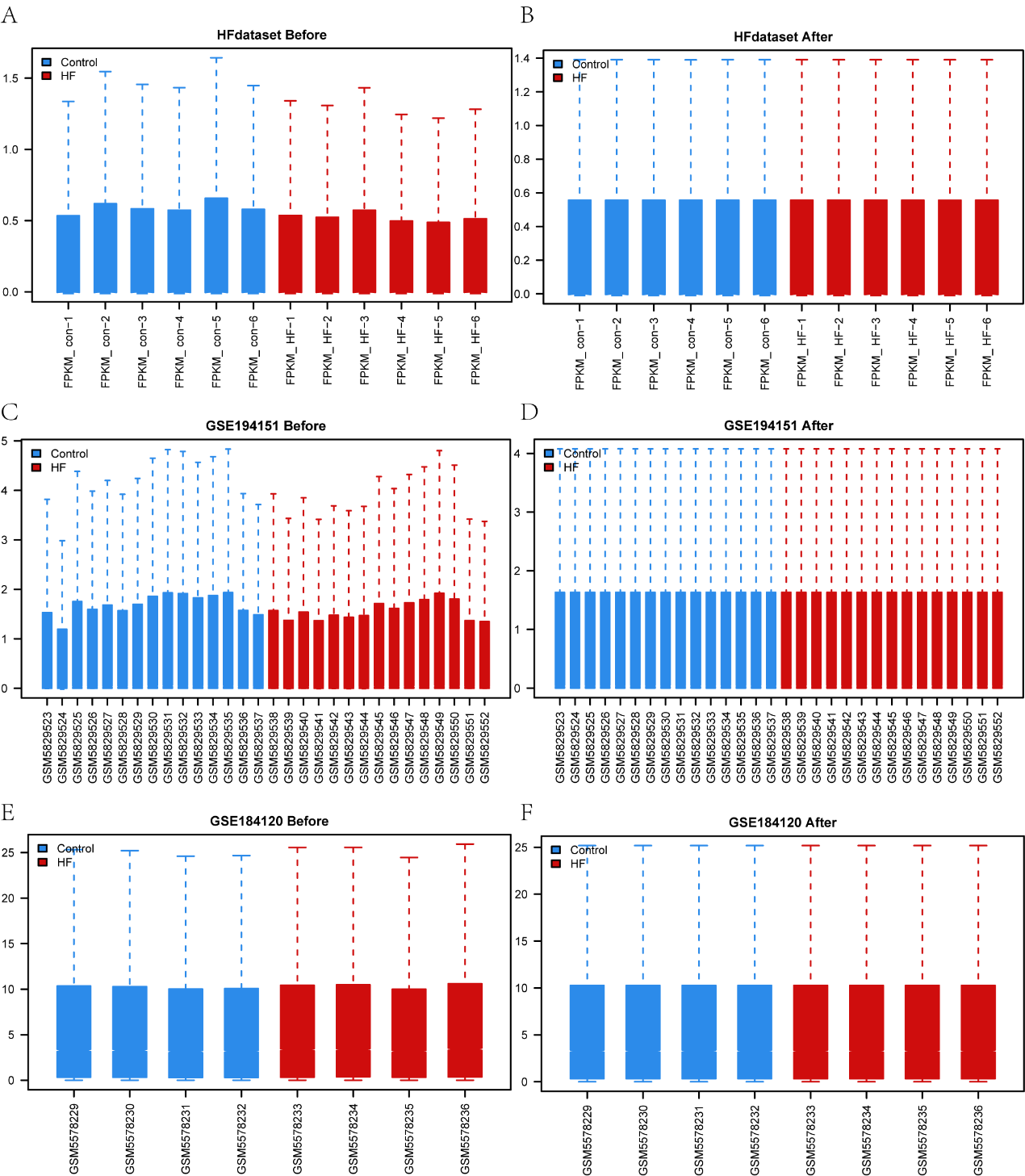


**Fig. S1 Normalization of the HFpEF datasets.** A-B. The boxplot before (A) and after (B) normalization of our own HF datasets. C-D. The boxplot before (C) and after (D) normalization of GSE194151 datasets. E-F. The boxplot before (E) and after (F) normalization of GSE184120datasets. HF, Heart Failure With Preserved Ejection Fraction.


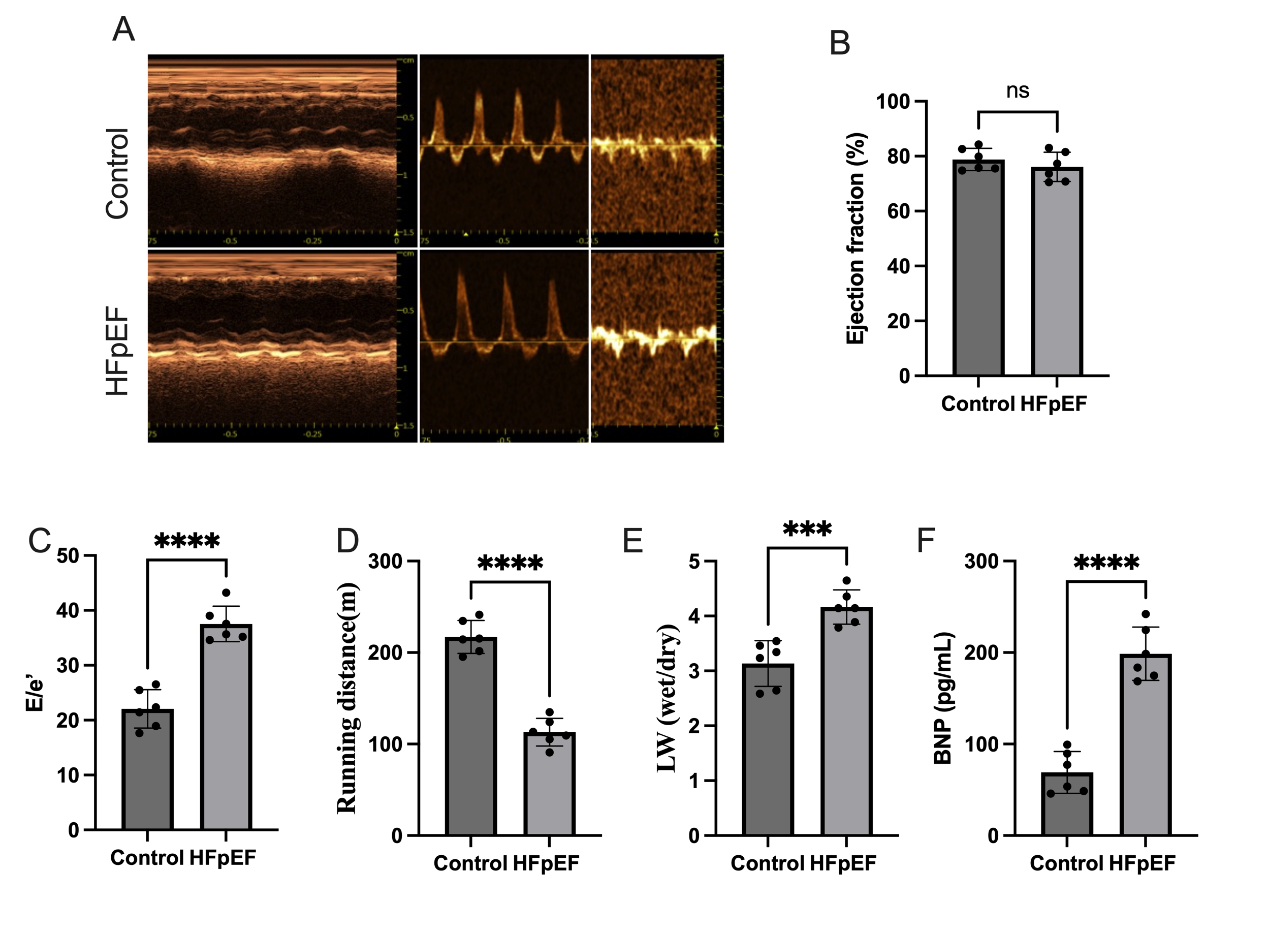


**Fig. S2 The successful induction of HFpEF mouse model.** A. Representative left ventricular M-mode echocardiographic tracings (left), pulsed-wave Doppler (medium) and tissue Doppler (right) tracings. B. The left ventricular systolic function was reflected by left ventricular ejection fraction. C. The left ventricular diastolic function was reflected by the ratio of E to e’. D. HFpEF mice showed reduced running distance. E-F. HFpEF mice showed increased ratio of wet to dry lung weight (LW) and plasma BNP levels (F). Not significant, ns.; ***, *P* <0.001; ****, *P* <0.0001. HFpEF, heart failure with preserved ejection fraction; LW, lung weight .


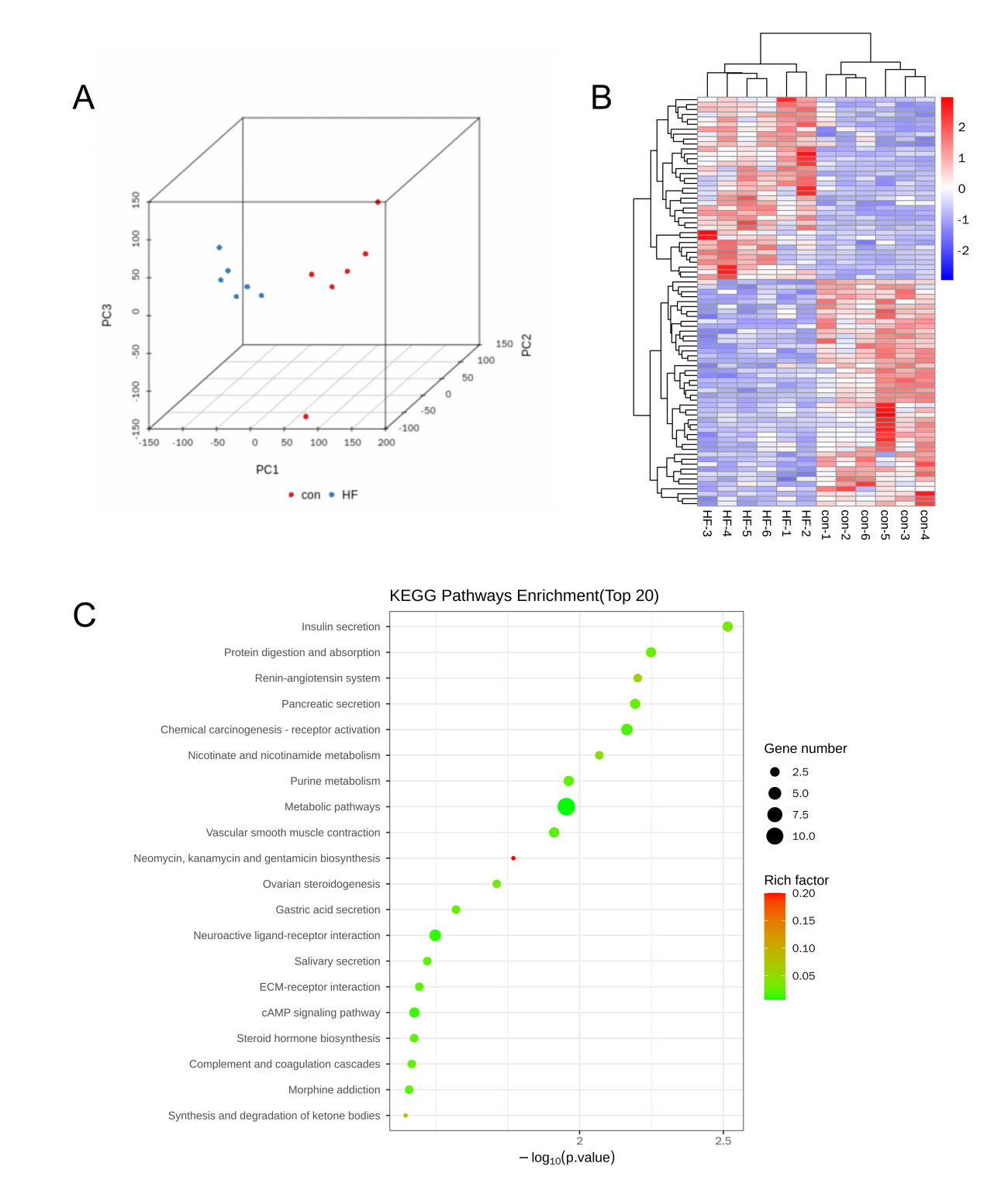


**Fig. S3** **The PCA and KEGG analysis of our own HF dataset.** A. The PCA analysis showed pronounced discrimination between HFpEF and Control hearts. B. The differentially expressed genes (DEGs) were displayed in the heatmap. **C**. KEGG pathway analysis was shown in bubble. HF, Heart Failure With Preserved Ejection Fraction.


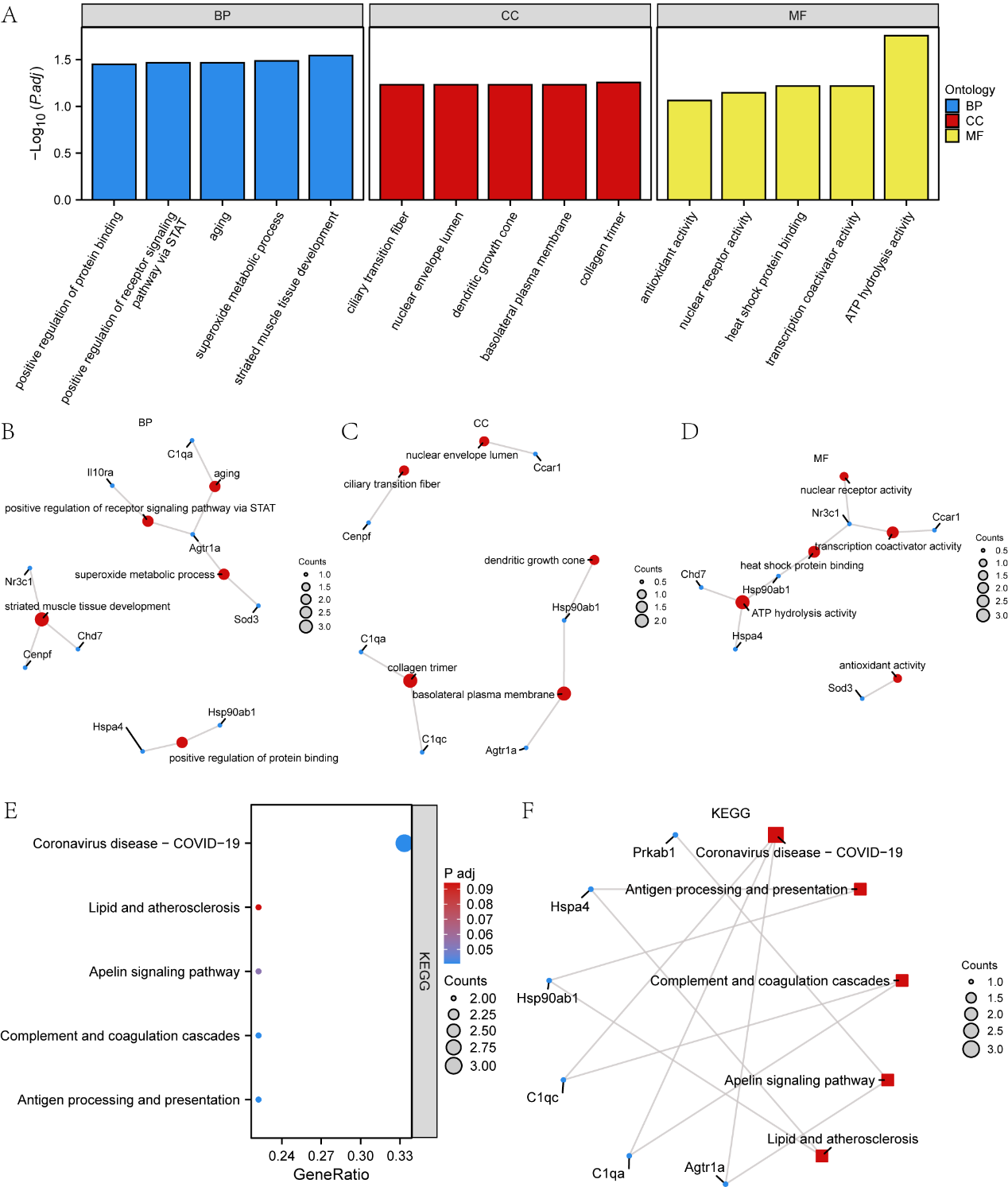


**Fig. S4 GO and KEGG analysis of ARDEGs.** A-D. The GO analysis of ARDEGs, including biological process (B), cellular component (C), and molecular function (D), respectively. E-F. KEGG pathway analysis of ARDEGs was shown in bubble (E) and ring network diagram (F). The x-axis represents different GO terms, the y-axis represents gene ratio enriched in relative GO terms in (A). The blue circle refers to gene and red circle refers to pathway in (B, C, D and F). and the color represents pvalue. The x-axis represents gene ration, the y-axis represents gene ratio enriched in relative KFGG terms in (E). GO, Gene Ontology; BP, biological process; CC, cellular component; MF, molecular function; KEGG, Kyoto Encyclopedia of Genes and Genomes; ARDEGs, Ageing related differentially expressed genes.


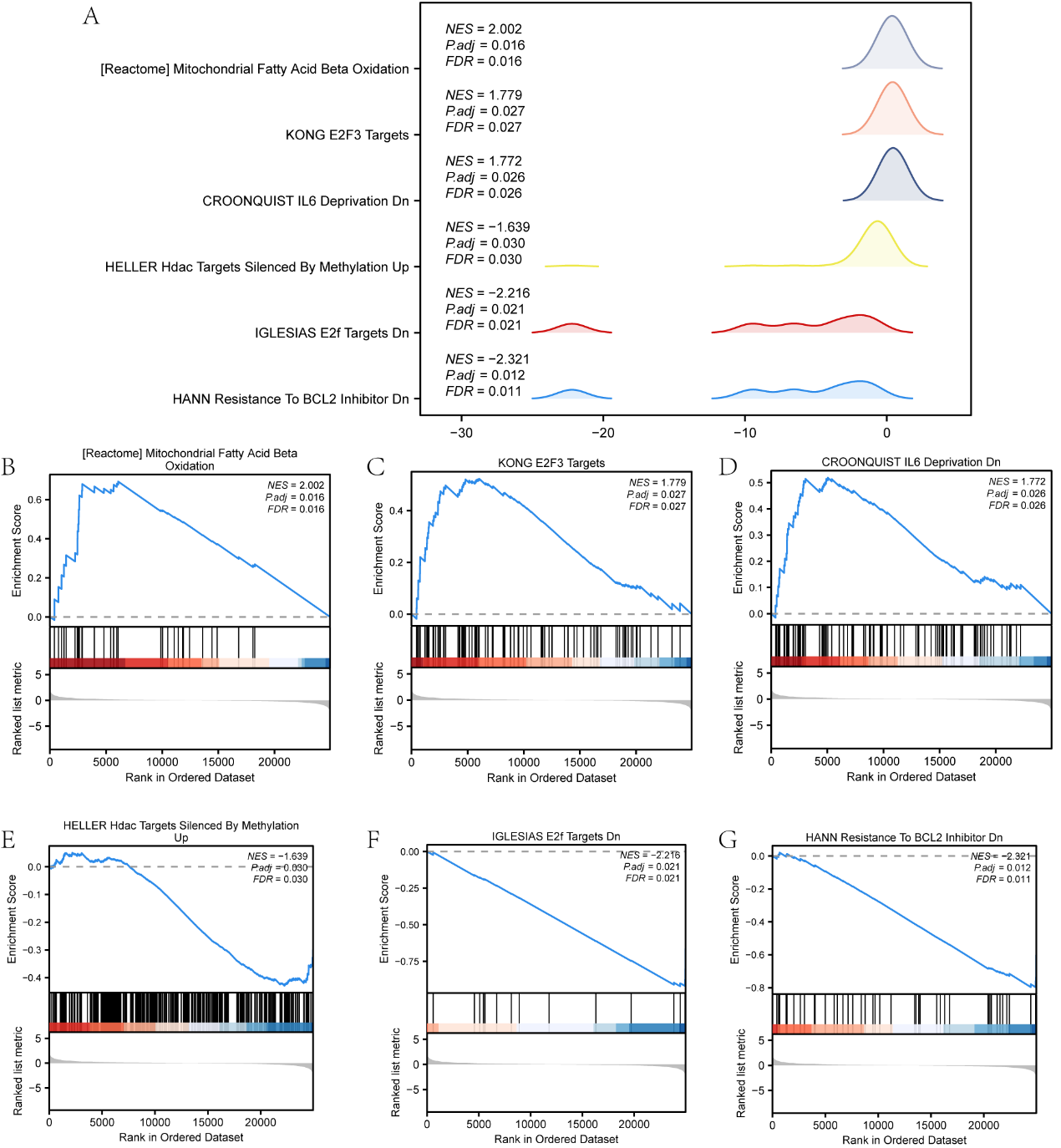


**Fig. S5 GSEA enrichment analysis of GSE194151 datasets.** A. The biological features of GSEA enrichment analysis were displayed in mountain maps. B-G. The Genes form different group (HF/Control) in GSE194151 datasets were significantly enriched in REACTOME_MITOCHONDRIAL_FATTY_ACID_BETA_OXIDATION (B), KONG_E2F3_TARGETS (C), CROONQUIST_IL6_DEPRIVATION_DN (D), HELLER_HDAC_TARGETS_SILENCED_BY_METHYLATION_UP (E), IGLESIAS_E2F_TARGETS_DN (F), HANN_RESISTANCE_TO_BCL2_INHIBITOR_DN (G) pathway. GSEA, Gene Set Enrichment Analysis; HF, Heart Failure With Preserved Ejection Fraction. Gene sets with P.adj< 0.05 and FDR q <0.05 were considered to be enrichment significant.


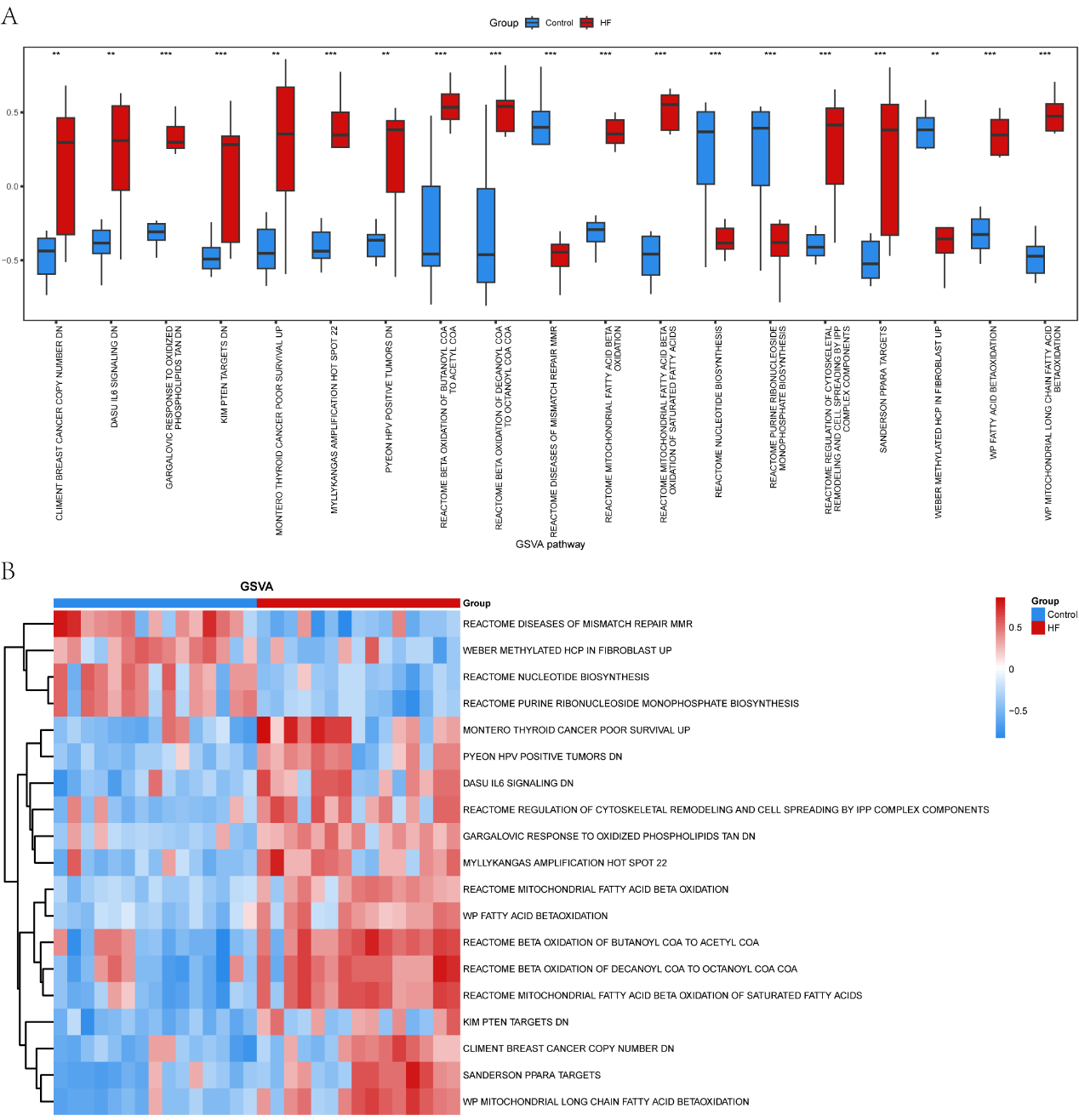


**Fig. S6 GSVA enrichment analysis of GSE194151 datasets.** A-B. Comparison chart (A) and heatmap illustrating the result of GSVA. *, P < 0.05; **, P < 0.01; ***, P < 0.001; HFpEF，Heart Failure With Preserved Ejection Fraction；HF，Heart Failure With Preserved Ejection Fraction；GSVA：Gene Set Variation Analysis. P.value < 0.05 and |logFC| > 0.50 were considered to be enrichment significant.


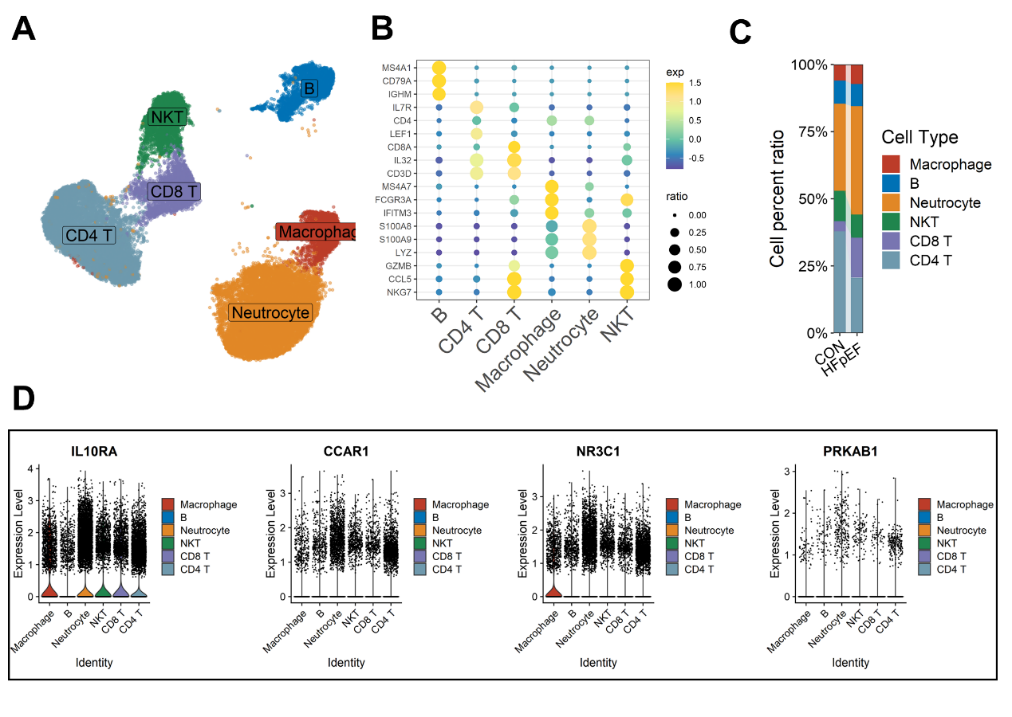


**Fig. S7 . The expression of ARDEGs in immune cells from peripheral blood of HFpEF patients.** A-B. Six types of different immune cells (A) were identified based on the maker shown in B. C. The proportion of different types of immune cell between groups. D. the expression of ARDEGs in different types of immune cells; HFpEF, Patients with Heart Failure With Preserved Ejection Fraction；CON，Healthy control.
