# Supplementary material for: Identification of ageing-associated gene signatures in heart failure with preserved ejection fraction by integrated bioinformatics analysis and machine learning
Source: Genes Dis. 2024 Dec 3;12(4):101478. doi: 10.1016/j.gendis.2024.101478 (PMC12053710; doi:10.1016/j.gendis.2024.101478)
Supplement: Multimedia component 2 [file mmc2.docx]

### Table 1. Heart Failure With Preserved Ejection Fraction set information list.

|  | **HF dataset** | **GSE194151** | **GSE184120** |
| --- | --- | --- | --- |
| Platform | / | GPL24247 | GPL25947 |
| Species | Mus musculus | Mus musculus | Rattus norvegicus |
| Tissue | Heart tissue | Heart tissue | sinoatrial node tissue |
| Samples in HF group | 6 | 15 | 4 |
| Samples in Control group | 6 | 15 | 4 |
| Reference |  | Sex differences in heart mitochondria regulate diastolic dysfunction | Mechanisms of Sinoatrial Node Dysfunction in Heart Failure With Preserved Ejection Fraction |

HF，Heart Failure With Preserved Ejection Fraction；GEO，Gene Expression Omnibus。
